# An Integrated Simulation Module for Cyber-Physical Automation Systems †

**DOI:** 10.3390/s16050645

**Published:** 2016-05-05

**Authors:** Francesco Ferracuti, Alessandro Freddi, Andrea Monteriù, Mariorosario Prist

**Affiliations:** 1 Dipartimento di Ingegneria dell’Informazione, Università Politecnica delle Marche, Via Brecce Bianche, 60131 Ancona, Italy; f.ferracuti@univpm.it (F.F.); a.monteriu@univpm.it (A.M.); 2 SMART Engineering Solutions & Technologies (SMARTEST) Research Centre, Università degli Studi eCampus, Via Isimbardi 10, 22060 Novedrate (CO), Italy; alessandro.freddi@uniecampus.it

**Keywords:** Contiki Os, COOJA simulator, cyber physical systems, hardware in-the-loop, Internet of Things, software in-the-loop, wireless sensor networks, WSN simulators

## Abstract

The integration of Wireless Sensors Networks (WSNs) into Cyber Physical Systems (CPSs) is an important research problem to solve in order to increase the performances, safety, reliability and usability of wireless automation systems. Due to the complexity of real CPSs, emulators and simulators are often used to replace the real control devices and physical connections during the development stage. The most widespread simulators are free, open source, expandable, flexible and fully integrated into mathematical modeling tools; however, the connection at a physical level and the direct interaction with the real process via the WSN are only marginally tackled; moreover, the simulated wireless sensor motes are not able to generate the analogue output typically required for control purposes. A new simulation module for the control of a wireless cyber-physical system is proposed in this paper. The module integrates the COntiki OS JAva Simulator (COOJA), a cross-level wireless sensor network simulator, and the LabVIEW system design software from National Instruments. The proposed software module has been called “GILOO” (Graphical Integration of Labview and cOOja). It allows one to develop and to debug control strategies over the WSN both using virtual or real hardware modules, such as the National Instruments Real-Time Module platform, the CompactRio, the Supervisory Control And Data Acquisition (SCADA), *etc*. To test the proposed solution, we decided to integrate it with one of the most popular simulators, *i.e.*, the Contiki OS, and wireless motes, *i.e.*, the Sky mote. As a further contribution, the Contiki Sky DAC driver and a new “Advanced Sky GUI” have been proposed and tested in the COOJA Simulator in order to provide the possibility to develop control over the WSN. To test the performances of the proposed GILOO software module, several experimental tests have been made, and interesting preliminary results are reported. The GILOO module has been applied to a smart home mock-up where a networked control has been developed for the LED lighting system.

## 1. Introduction

**Figure 1:**
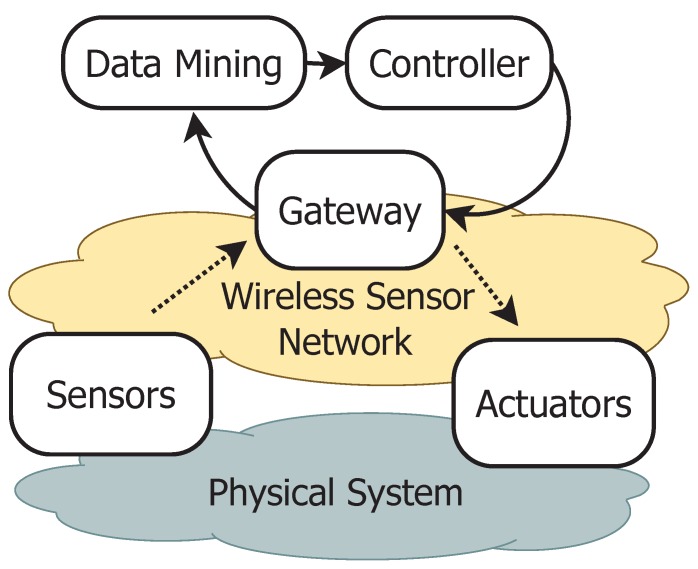
The architecture of the cyber-physical wireless control system.

A Cyber Physical System (CPS) integrates elements, such as sensors, actuators, embedded computers or microcontrollers and computational engines, which are networked to sense, monitor and control physical processes [1,2]. The CPS paradigm can be considered as an evolution of the Internet of Things (IoT), since they both share the same basic architecture. However, a CPS presents higher merging capabilities between physical and cyberspace elements, which are obtained in a two-step feedback loop. In the first one, the computational part of the cyberspace acquires data coming from the real system through the sensor network and uses the data to elaborate and manage the control policies. In the second one, the computational engine schedules the control decisions according to the feedback control loop model and acts on the physical world by using analogue/digital outputs of the hardware devices in order to obtain the desired changes [3,4]. Over the past few years, CPSs had a profound impact on different engineering fields due to their multidisciplinary nature and thanks to the usage of wireless technology. In particular, several CPS solutions are employed in industrial automation [5] (e.g., process supervision, production lines, *etc.*), the automotive industry [6,7], body area networks [8] and environmental scenarios (e.g., smart homes, smart grids, *etc.*) [9,10,11]. In this context, the addition of control capabilities to the Wireless Sensor Network (WSN) paradigm is called Wireless Sensor and Actor Networks (WSANs) or Wireless Network Control System (WNCS), whose differences have been analyzed by Akyildiz *et al.* [12]. The control system schedules advanced control actions that allow the sensor nodes and actuators (see Figure 1) to interact directly with the physical process through Digital-to-Analogue Converter (DAC) output.

These wireless control systems may bring several advantages in terms of installation complexity reduction, lack of wiring and its related costs, enhanced system reconfiguration capability, and so on. However, in this context, critical challenges need to be solved before wireless control may overcome typical negative perceptions. In particular, the higher concern is about the quality of service, low energy consumption, process stability, which may not always be guaranteed, packet transmissions problems and high probabilities of packet losses. The integration of these aspects, nowadays a consolidated reality for cabled networks, needs to be performed by enhancing batteries life [13], management of delays, package delivery, real-time, security, *etc.* [14,15,16]. Networked control system architectures and their advantages over traditional control techniques have been recently introduced by Yang [17]. The physical delays between reading a sensor value and sending a control signal through a networked-based systems have been pointed out by Chow and Tipsuwan [18]. Moreover, the authors analyze the challenging problems related to the packet data loss during transmission with multi-hop routing techniques. Melodia *et al.* [19] introduced a new location management algorithm able to minimize energy expenditure, while controlling the delay of the data-delivery process based on power control.

With an ever-growing evolution of sensors, actuators, wireless antenna, processors and, in general, hardware, modern CPS structures require many resources in the design stage [20]. In this context, a reliable simulation process becomes an essential step to provide good designing results in a short time and in a cost effective way [21,22]. Although they play such a crucial role, not many open source network simulators have been developed for CPSs rapid prototyping. Furthermore, only a part of them is capable of properly emulating actor-sensor nodes (*i.e.*, motes) with their own hardware specifications. Among them, there is an open source set of designing tools for embedded real-time control systems developed by the Institute for Software-Integrated Systems (ISIS) [23]. A Wireless Cyber-Physical Simulator (WCPS) has been introduced in [24], which integrates Simulink and the TOSSIM (TinyOS SIMulator) and has been employed to simulate the physical systems [25]. Unfortunately, TOSSIM has several limitations, since it provides support only for the the MICAZ hardware platform and the Tiny Operating System (TinyOS) [26].

**Figure 2:**
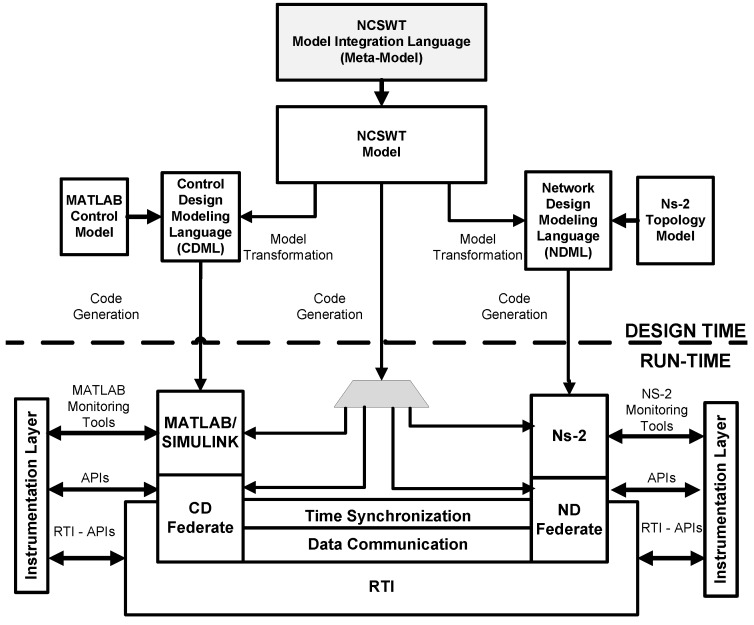
NCSWT tool chain [27].

**Figure 3:**
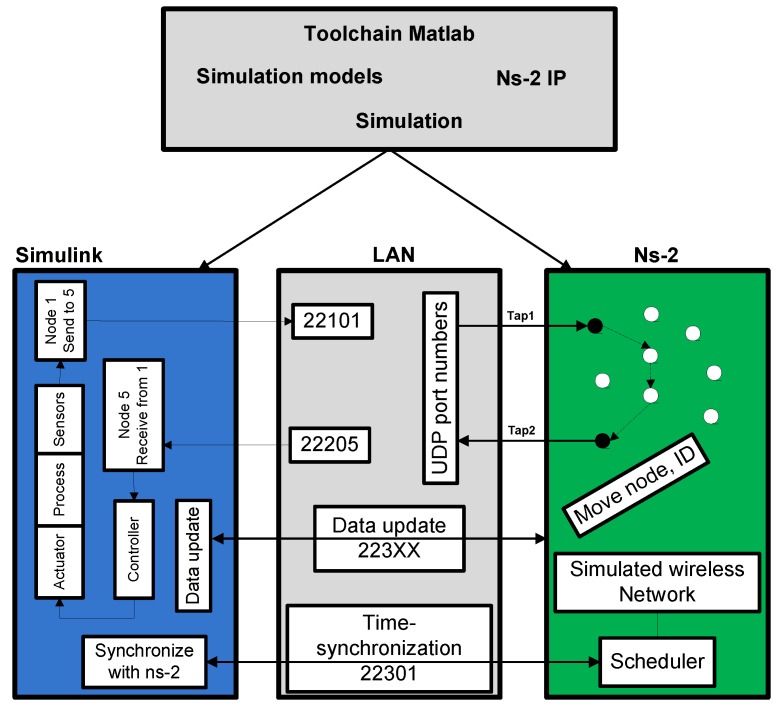
PiccSIM tool chain [32].

In this context, Eyisi *et al.* [27] have proposed an evaluation tool of networked control systems called Networked Control System Wind Tunnel (NCSWT). It integrates MATLAB/Simulink and Network Simulator series 2 (NS-2) according to the High Level Architecture standard (HLA). The framework provides both data communication and time synchronization features for heterogeneous simulations (see Figure 2). The “Platform for integrated communications and control design, Simulation, Implementation and Modeling” (PiccSIM) composed of Simulink and NS-2 has been proposed by Nethi et al. [28]. The communication between Simulink and NS-2 is based on UDP packets. The protocol is exploited for sending and receiving sensor data, for node positioning and time synchronization (see Figure 3). N2 simulators, well known in the literature, *i.e.*, Simulink and COOJA, are integrated in “GISOO”, which is a new virtual testbed for wireless networked control systems developed by Aminian et al. [29]. GISOO is based on the cross-level simulator COOJA that enables one to simulate, at the same time, the medium level, the network level and the operating system level [30]. Moreover, its integration in Simulink allows one to extend the three levels by adding a new physical system modeling level [31].

**Figure 4:**
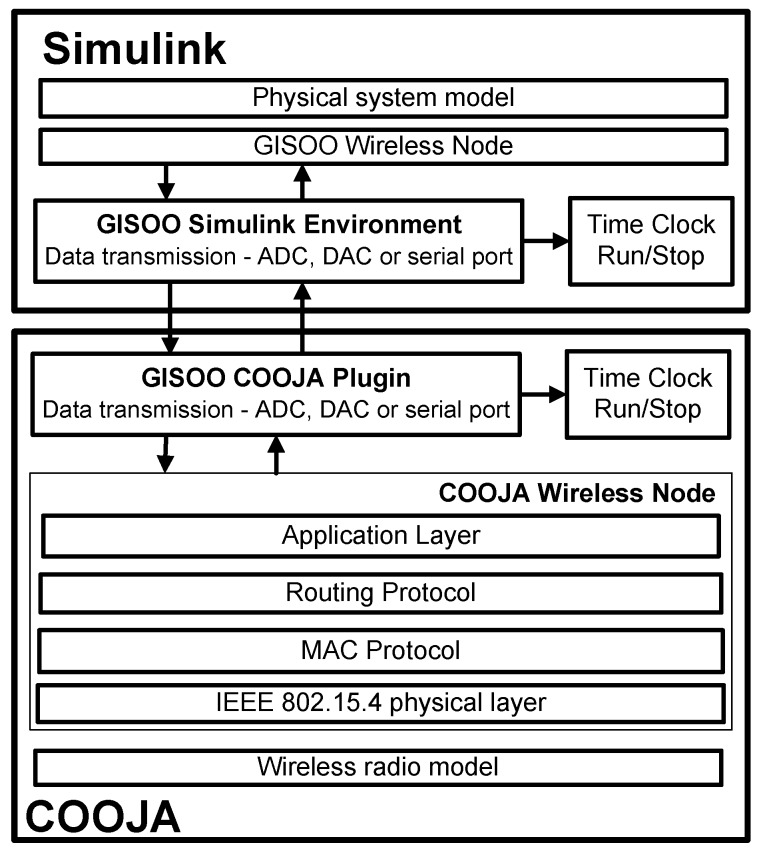
The architecture of GISOO.

The GISOO architecture is shown in Figure 4, where emphasis is given to the UDP communication and time synchronization.

Although the simulator is released with an open source policy, high flexibility and built in integration with the MATLAB/Simulink environment, the key CPS features, *i.e.*, the physical connection and interaction with the processes to be controlled, are completely missing. Furthermore, the interaction with the motes in the simulation environment is very difficult, and it is not possible to stimulate them with consistent analogue input/output values for closed loop policy simulations.

In this paper, we design a software module for control purposes over WSNs in order to cope with the challenging CPS simulation limits described above. The module is based on the GISOO plugin and in particular, it integrates the LabVIEW environment and COOJA. The developed module is called “GILOO” (Graphical Integration of Labview and cOOja) and allows one to design and debug control policies, in real or simulated scenarios, through the LabVIEW environment and National Instruments hardware, such as the PXI platform, CompactRio, *etc.* [33]. Since for the Sky mote [34], the analogue output control is missing in Contiki Os [35], in this work, we have developed the Contiki Sky DAC driver, as well. The driver has been tested in the COOJA Simulator with the new Advanced Sky GUI [36,37], in order to implement the control over the wireless network [29,31]. The focus on the sky platform is due to its excellent features and performance; in fact, Telosb/Sky is one of the most important all-in-one wireless sensor nodes used in WSN research and real applications composed of a power source, transducers, processor and radio transceiver.

The following steps have been carried out in order to design and test the proposed software module for CPS simulation:

1. The GISOO interface between COOJA and Simulink via UDP communication and time synchronization has been analyzed.
2. LabVIEW has been used to design the necessary software interface.
3. A hardware layer interface, compatible with Texas Instruments platforms, has been realized for CPS prototyping.

In this paper, Section 2 and Section 3 introduce the architecture of the GILOO module, how it integrates with the GISOO plugin and the structure of the virtual environment which has been developed by using LabVIEW. Section 4 describes the Sky DAC driver architecture. In Section 5, the COOJA Simulator, the “cross-levels” concept and the designed software integration for the Advanced Sky Module are presented. Section 6 reports the preliminary experimental tests and results. Section 7 provides remarks and future works.

## 2. Architecture Concept Layout

The developed software module, namely GILOO, permits one to integrate a physical system with a cyber system, thus granting computational capabilities for validating and debugging CPSs in an innovative way. The external connection of the cross-level simulator COOJA with LabVIEW permits one to open new prototyping scenarios, like simulating control laws over wide and heterogeneous WSNs, or to use either physical, or virtual processes, or plants. An interesting aspect of LabVIEW is that, in addition to the data acquisition, instrument control and modeling capabilities, it can be used to implement control architectures through FPGA, SCADA, PLC, *etc*. This is important in CPSs, since it extends the COOJA levels, by adding a level of interaction with the process to the already existing network operating system and code instruction set levels. In detail, the sensor motes are used to feed the process information to the control law, which elaborates the information and generates the action to be performed by the actor motes. Topology, wireless medium characteristics and sensor hardware can be modeled thanks to COOJA, while the physical process can be designed thanks to LabVIEW, and the control law can be designed either in COOJA or in LabVIEW according to the desired control objectives. The integration achieved through the developed module permits one to substitute one or more virtual components with real components, thus providing a fully-integrated rapid prototyping environment for CPSs.

Let us consider a typical use case of a CPS in which the control action is closed through a WSN, and GILOO is used for rapid prototyping and the control law testing. The overall closed-loop system is made of:

- the physical process, including the motes, which can be divided into:
  - sensor motes for acquisition and reading data from the physical field;
  - actor motes for implementation of distributed or centralized control techniques and direct interaction with the physical-devices;
  - repeater motes, which extend the wireless communication distance and permit one to supervise the entire plant.
- the WSN, operating according to the IEEE 802.15.4 radio communication standard;
- the control hardware, performing the desired control law (e.g., SCADA, PLC, *etc.*).

In the first step, the process is modeled in LabVIEW; the WSN is modeled in COOJA with the same number of actors as motes (required for controlling the process); and the control law is designed in either in COOJA or LabVIEW. The virtual sensor motes are connected to the virtual sensor motes of the COOJA simulator, which gather the data to be processed by the control law. The control law generates the command signals to be sent to the virtual actor motes through the related COOJA virtual actor motes. GILOO acts as a bridge between the process and COOJA, by using the external communication modules of National Instruments, on the one hand, and the developed GILOO LabVIEW blocks, on the other. In this way, the designed control law is tested taking into account the process dynamics and disturbances, the sensor hardware, the network medium and topology. Once the desired results are achieved, each block can be substituted with a physical one: the control law can be implemented into the desired control hardware; the simulated WSN can be replaced by a real WSN with the same topology, medium and motes (including the firmware); while the virtual process is substituted with the physical one.

The main contribution of our work is the development of the GILOO software module, which extends COOJA by providing a fourth level of simulation, namely the connection with the process. Unfortunately, at the present state, one of the most widespread research motes, *i.e.*, the Sky mote [34], is not fully supported in COOJA, since actor motes cannot generate analogue outputs. This means that a full exploitation of the four-level simulator is not possible, since the control action cannot be simulated. This is the reason behind the second main contribution of the paper, namely the Sky DAC driver (together with the GUI module): the developed solution permits one to simulate the Sky analogue control outputs, thus fully exploiting the four-level simulation design for rapid prototyping. Moreover, the structure of the proposed DAC driver can be used for reference design in other motes, as well.

In the next two sections, we will describe in detail the architecture of the GILOO software module, the Sky DAC driver and relative GUI.

## 3. GILOO Software Module

**Figure 5:**
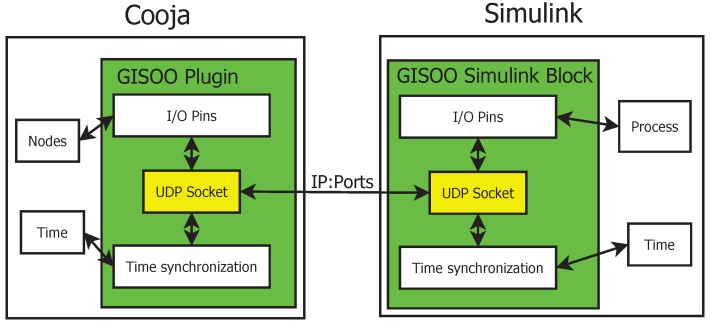
Communication between GISOO and COOJA.

The proposed GILOO software module is built over the GISOO plugin, which is developed by the KTH (Kungliga Tekniska Högskolan) Royal Institute of Technology, and permits one to connect COOJA to Simulink, as shown in Figure 5. Before explaining the blocks developed in the LabVIEW environment, it is important to describe the functional diagram and how the GISOO module works to implement the outside connection with COOJA. GISOO can be decomposed into two principal elements: the COOJA plugin and the Simulink blocks.

- The COOJA plugin manages the ADC, DAC, I/O, serial, *etc.*, of the emulated nodes through the instruction set of the microcontroller and the state machine of the simulation environment, in order to preserve both time synchronization and data consistency between the two simulators. The interaction of COOJA with Simulink is managed by exchanging special UDP packets. A formatted frame within a custom protocol realizes a complete set of information, which is needed to ensure that the process operates correctly.
- The Simulink architecture blocks permit one to create a bidirectional communication channel with COOJA, to control the analogue and digital pins of the simulated nodes, both for the input (ADC) and the output (DAC). Simulink transfers data and time synchronization information by using UDP (reader and receiver) blocks, which manage packets on a specific port.

### 3.1. GILOO Architecture

As previously described, GILOO is the key point of the architecture and the main contribution of the proposed work. It is made of two principal elements: the Virtual Instruments (VIs) and the GISOO plugin. The VIs are the constituting blocks of a program written in LabVIEW, are similar to the functions/routines of other programming languages and model the real behavior of a physical device. They include the front panel, which acts as the Graphical User Interface (GUI) to manage the user interaction events when the program is in the run state, the block diagram, which contains the source code in a graphical form, operators and structures from built-in LabVIEW VI libraries, and the connector panel for joining a VI to another VI. The GISOO plugin integrated in the COOJA simulator listens to any function call generated by the native ADC pins, DAC pins and UART port pins (TX and RX) in the real wireless sensor nodes [29].

The GILOO-LabVIEW library includes the blocks for managing both time synchronization and data communication. Serial messages, their bytes conversion and checksum have been elaborated in a dedicated sub-VI routine.

### 3.2. Communication Messages

GILOO is based on the GISOO software plugin. This module is a “mote plugin type” like the Advanced Sky GUI, which means that the co-simulation starts only in the case that, on a selected mote, a GISOO interface is initialized, so that a UDP socket is opened, initialized and starts to listen for data from the associated mote in LabVIEW.

The UDP protocol is intrinsically unreliable, since it does not implement a handshake mechanism to establish a connection between the sockets or a mechanism to retry after transmission failures or data corruption (as TCP does). This implies that there is no guarantee that a UDP packet reaches its destination, that the datagrams are received only once or their order is preserved across the network. Moreover, UDP has a message length limit of 65,536 bytes, but this limit is relevant only for applications that require a large amount of packets to be exchanged, while it grants performance-intensive functions and low RAM workload in applications like WSN simulators.

Due to the absence of the communication control and the connection-less service provided by UDP, no explicit connection is established between client and server for data transmission. A client has to listen to a specific UDP port and receives all of the broadcast data carrying its address. COOJA manages the network traffic by sending packets according to the read/write requests of the emulated node inputs/outputs.

When LabVIEW receives a new packet, it checks for the correct format and the data integrity, analyzes the payload by searching for the special bytes or commands and, finally, dispatches the analogue/digital values and settings to the correct address. The communication message has a structure that can be decomposed into four parts:

- Simulation Time
  This defines the simulation time of COOJA, and it is used to manage the synchronization with LABVIEW.
- Mote Pin ID
  This represents the unique identifier of the mote in LabVIEW, and it is a required setting to correctly redirect the request of a node in LABVIEW (it selects both the pin and the mote).
- Actuation value (uValue)
  It refers to the DAC value and its related mote pin. This value, coded with three bytes, is contained from the eighth to eleventh position of the message.
- Serial Data
  To permit data exchange in serial communication, sixteen bytes of the UDP message have been reserved, and if the serial data are more than serial bytes allocation, a segmentation technique is required. The structure of the serial message is based on the TinyOS specification, so that a mote can support multiple UART packet formats at the same time [38]. In addition, the serial bus can send or receive only messages formatted like the Serial ActiveMessageC frame, and it is not possible to exchange raw data from motes. This specification defines a platform-independent active message layer, which works at the high level of the serial communication stack of TinyOS.

### 3.3. Time Synchronization

Time synchronization between the two simulators is realized as follows. When the two software programs are started, then the LabVIEW simulation time is paused until a COOJA reading request is generated: this means that COOJA stays in a run state at normal speed. This event occurs every time an ADC reading, DAC writing or serial communication is requested in the mote. When a message is received by LABVIEW, then the simulation is resumed, while COOJA simulation is paused. After the needed elaborations, LabVIEW sends the data back to COOJA, and it pauses the simulation time. When COOJA reads the packet containing both data and command, the information is made available to the motes, and the simulation time is resumed. Generally, in LabVIEW, it is not possible to manage the pause/resume of simulation in run-time (unlike Simulink); thus, the following structures are required: one “flat sequence structure”, composed of two sequentially executed frames, a No OPeration (NOP) and two “while loops”. The first frame handles the time within COOJA, while the second frame handles the program interacting with the motes in COOJA.

## 4. Sky DAC Driver

The hardware devices of a wireless sensor network, generally, are composed of sensors and transceiver modules, which communicate with the firmware by using a specific software structure, namely the driver. Although the operation principle of different types of sensors could vary, the hardware interface and hardware interaction are achieved by using analogue or digital connections. For each type of hardware connection and device, a software interface is needed. We focus our attention now on one of the most used motes for research purposes, *i.e.*, the Sky mote, providing the details of the Sky DAC driver integrated in Contiki OS. By using the proposed driver, it is then possible to simulate a Sky-based mote in COOJA, including actor motes with analogue output, as well.

### 4.1. Contiki OS Kernel

The hardware sensors drivers, in Contiki OS, are written in compliance with the main sensor process interface specifications, so that this process can schedule and launch the internal routines [35]. Data acquisition generally starts by calling specific process sensors, for example the process to measure humidity, temperature, and so on. These processes are controlled by the sensor scheduling process, namely the *sensor_process*, and started at Contiki boot. This process manages two principal arrays as described below:


***const extern struct sensors_sensor sensors[]*** - Every sensors is associated with a *sensors_sensor struct*, a sensor’s pointers array, and each pointer is referred to a specific function in the driver files:
     struct sensors_sensor {
      **char** * type;
      **int** (* value ) (**int** type );
      **int** (* conf igure ) (**int** type , **int** value );
      **int** (* status ) (**int** type );
     };
***extern unsigned char sensors_flags[]*** - The attached sensors are identified by flags and are used when the function sensor_changed() is called.
		

In order to integrate the Sky DAC driver into Contiki OS, it is important to follow the operating system guideline specifications used for the ADC driver, so the user program can invoke the Contiki’s functions for the sensors reading, like SENSORS_DEACTIVATE(...), SENSORS_ACTIVATE(...) and xxx_sensor.value(...). In our approach, we use similar names both for the reading and writing functions.

### 4.2. Driver Implementation

**Figure 6:**
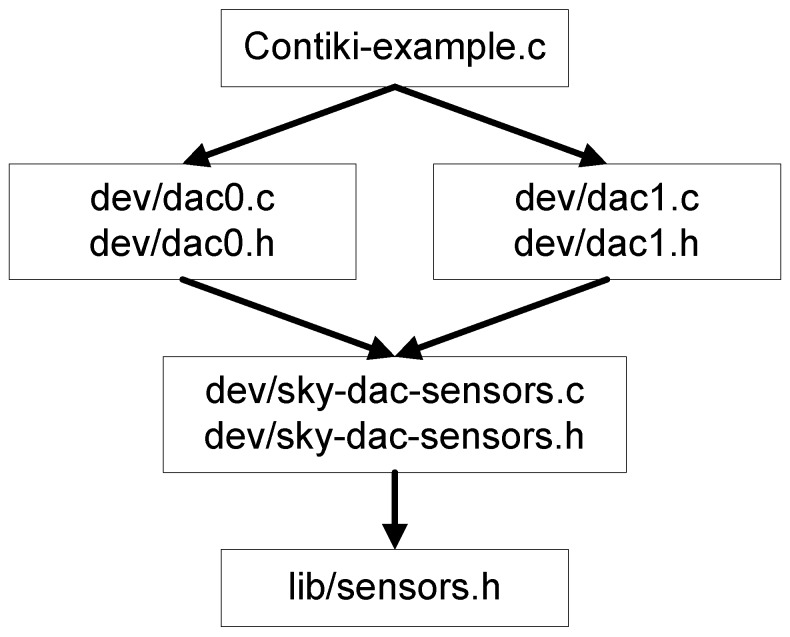
An illustrative example of the Sky DAC drive architecture.

The Sky mote DAC driver for Contiki OS proposed in this work is based on a TI MSP430F1611 microcontroller used for the Sky mote. The MSP430F1xx family by Texas Instruments is an ultra-low power microcontroller that has two standard DAC ports (DAC0 and DAC1) associated with P6.6 and P6.7 respectively, and two setting registers, called DAC12_0CTL and DAC12_1CTL. The configuration of a DAC value is done by the digital values and a reference $V_{ref}$ voltage, which sets the analogue output scale. The ports P6.6 and P6.7 of the Sky microcontroller are both for ADC and DAC; thus, it is not possible to read and write simultaneously. Moreover, in *lib/sky-sensors.c*, the *cc_inline* functions available, used to manage the start and stop of reading operations, cannot be modified nor used. Figure 6 illustrates the proposed architecture. The DAC driver for each port consists of two dedicated files, *dac0.c* (or *dac1.c*) and *sky-dac-sensors.c*, together with their header file. The port DAC1, for example, has the *dac1.c* file, which consists of the following definitions to control the reference voltage, the output channel and the register name:

#define OUTPUT_CHANNEL (1 << INCH_7)
#define INPUT_REFERENCE SREF_0
#define DACDAT1 DAC12_1DAT

The configuration parameters, the setting of the DAC value and the retrieval of the DAC status are managed by the following functions:

***static int value***(***int input***) - The variable *input* is used to change the value in the DAC data register.
***static int configure***(***int type, int c***) - The configuration parameters are the OUTPUT_CHANNEL and INPUT_REFERENCE, the SENSORS_ACTIVE or SENSORS_DEACTIVATE information and the status value of the DAC (*i.e.*, on or off).
***static int status***(***int type***)
		

Instead, the file *sky-dac-sensors.c* contains the following *cc_inline* functions:

***static CC_INLINE void startDAC***(***void***) - This function manages the start of DAC functionality. The procedure to enable DAC0 registers differs from that of DAC1 registers. A special function has been included in order to control the port selection.
***static CC_INLINE void stopDAC***(***void***) - This function turns off the reference voltage and stops DAC operations. It may be used to operate a sensor configuration change.
		

## 5. Advanced Sky GUI Module

COOJA is a wireless sensor network simulator used to emulate a large number of wireless sensor nodes running the application based on Contiki or TinyOS operating systems. Due to the flexible architecture, it integrates three simulation levels, the code level, the medium level and the network level. This enables the use of heterogeneous platforms, new programming approaches and the simulation of deployable code. The emulation of chip code level is done by the MSP430 Simulator (MSPSIM): it allows one to simulate the MSP430 instruction set in order to improve the rapid prototyping and to reduce the debugging time. Although different platforms based on the MSP430 chip can be simulated in COOJA, some features are not supported, and the mote GUI manages only a few properties. In detail, the virtual motes created with COOJA present the problem that the simulated analogue ports generate random values. Basically, this would make the introduction of the fourth level of simulation (the application level) useless, since it would not be possible to check if a control technique is acting on the specific operation, due to the absence of consistent feedback data. Moreover, there is an absence interaction of the external environment with the motes pins, and the movement of a mote within the COOJA can be realized only using the mouse or, if the path is known, using a “Mobility Plugin” [39].

**Figure 7:**
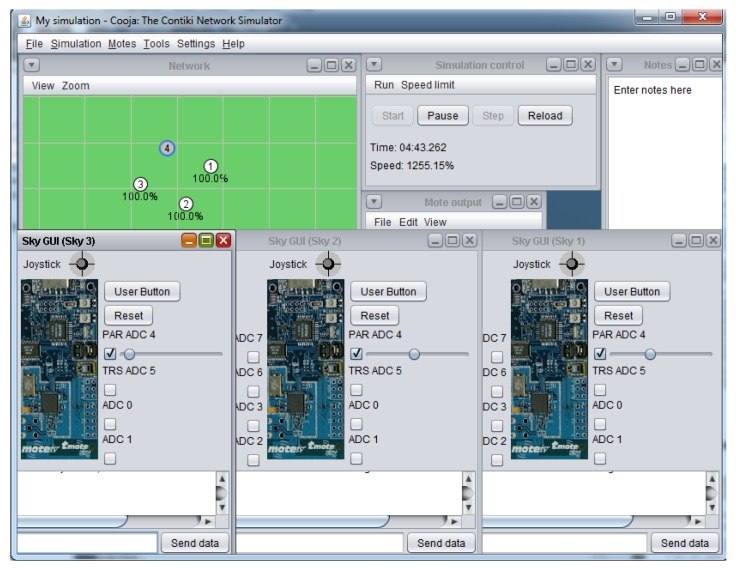
Advanced Sky GUI application.

**Figure 8:**
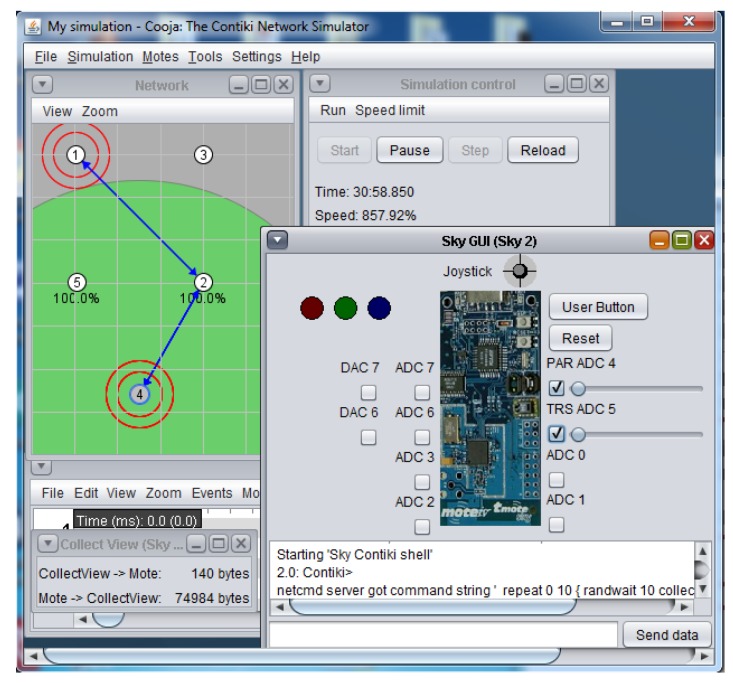
Advanced Sky GUI.

The “Advanced Sky Graphical User Interface”shown in this work integrates the hardware interfaces of the sky mote in only one software module, as shown in Figure 7 and Figure 8. The Advanced Sky GUI presents two indicators for the DAC output values, eight indicator sliders to vary the ADC input values, and provides a movement console in order to shift the Sky-mote inside the virtual environment.

**Figure 9:**
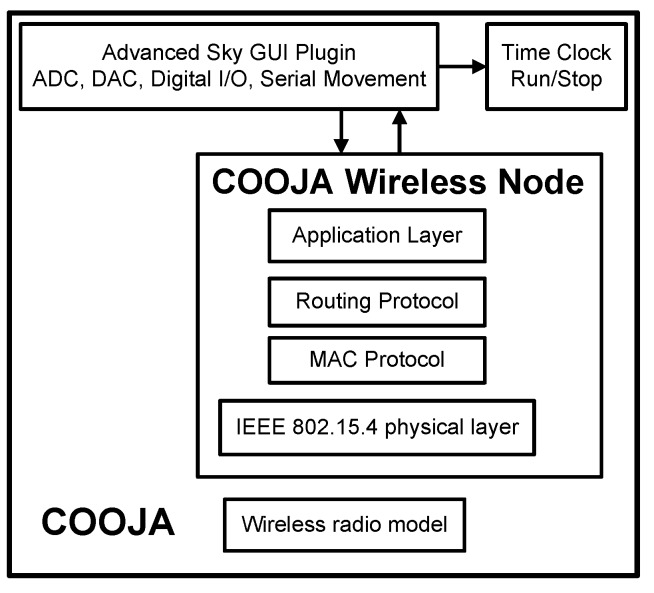
Advanced Sky GUI architecture.

The standard MSPSIM version integrated in COOJA manages the emulation of the digital input/output and analogue input (ADC), but does not implement the functions for managing the analogue output, which is the one of the most important features for control applications. The Advanced Sky GUI module used in this context implements a special version of MSPSIM with DAC12 integration, developed by KTH University. As shown in Figure 9, the architecture outlines the “mote plugin type”, a specific interface module that COOJA can manage.

The turns of the node settings are not properties of the simulation environment, but of the nodes themselves; thus, in runtime, the interface module is dynamically loaded only when the user interacts with the mote and explicitly requests, through the mouse, to open the Sky interface. The analogue input is controlled by the slider, which generates a scaled value with the same resolution of the Sky ADC ports, while a 12-bit voltage value is provided by the DAC outputs. The following code lines provide the connection between the emulated I/O manager by MSPSIM and the analogue ports:

((ADC12)skyMote. getCPU().
 getIOUnit(“ADC12”)). setADCInput(...);

((DAC12)skyMote. getCPU().
 getIOUnit(“DAC12”)). setDACOutput(...);	  
	  
while the interaction for the mote movement within the environment through the COOJA interfaces are provided by:

**double** x = mMotePosition. getXCoordinate()	
   + xIncr / speedMove;  
**double** y = mMotePosition. getYCoordinate()
   + yIncr / speedMove;
	  

In order to enhance the usability, a virtual controller has been designed that permits one to change the direction and the movement velocity of the selected node.

Until now, the Contiki Sky DAC driver has been developed and tested in the COOJA Simulator with the new Advanced Sky GUI. The Advanced Sky GUI plugin is already available and can be downloaded from the link reported in [36].

## 6. Experimental Results

In this section, we present the experimental results obtained by using the GILOO software module and the Sky driver and GUI in two different application scenarios.

GILOO has been tested for the networked control of an LED lighting system for indoor applications. This scenario has an architecture that is similar to that of smart homes or industrial automation systems; thus, it can be easily extended for controlling systems with a much larger number of inputs and outputs over a communication bus [40]. In detail, GILOO has been used for rapid prototyping and debugging of the wireless LED lighting control, whose objective was that of keeping the illuminance level at a desired set point value. In this case, we did not use the analogue output generated through the Sky DAC driver, but opted for a multi-port commercial converter that is commonly used in industrial applications. This work is based on serial communication of the GILOO module, but it is possible to use with the ADC and DAC functionality. Furthermore, a comparison analysis between GILOO and the real system has been done to confirm the correct behavior of the proposed solution.

**Figure 10:**
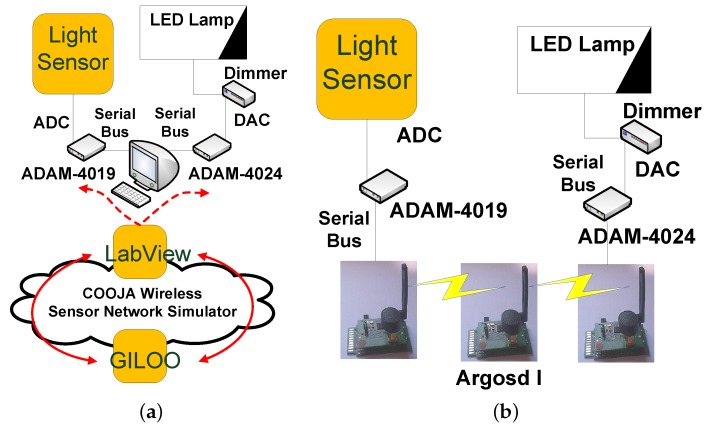
Application architecture: co-simulation and real scenario. (**a**) rapid prototyping architecture; (**b**) real architecture.

The system architecture, shown in Figure 10, is composed of:

- GL LED Panel, 1200 mm × 300 mm, cold light, 50 W of nominal power and light intensity of 4350 lm.
- DLC1248 single channel LED dimmer, driven by a 0–10-V analogue signal.
- ADAM-4019 universal analogue input module by Advantech, a eight-port ADC-to-serial converter. The ADAM Series covers a set of sensor-to-computer interface modules that contain a microprocessor, use a set of ASCII format commands, an RS-485 bus and can be remotely controlled.
- ADAM-4024 analogue output module by Advantech, a four analogue output and four isolated digital input port DAC-to-serial converter. It uses ASCII format commands, an RS-485 bus and can be remotely controlled.
- The ANS-LUX-A lux meter by NESA for acquiring a high accuracy level of the environment illuminance.

**Figure 11:**
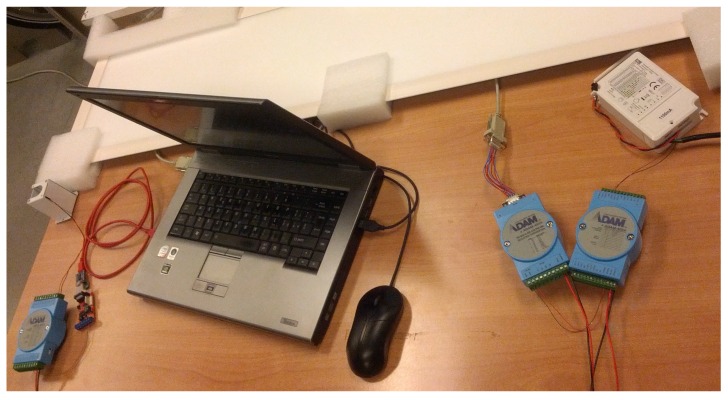
Experimental application plant.

The environment illuminance is the reference variable of the system, which must be acquired using the ADAM-4019, the ADC-to-serial converter and read by the serial port of the wireless mote. In this architecture, the LabVIEW program works as a gateway through the GILOO module and redirects data from the ADAM modules to COOJA and *vice versa*. The ADAM-4019 sends the data acquired by the lux meter to LabVIEW via serial bus, which sends a UDP packet to the simulated motes in COOJA (see Figure 11).

**Figure 12:**
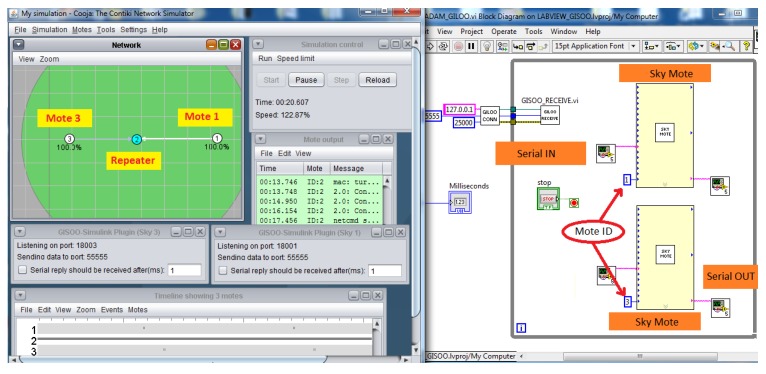
Integration of GILOO module in the control application.

COOJA has been used to design the WSN over which the control action is generated. The WSN is composed of three nodes: the first node reads the serial frame and sends it to the second node; the second node acts as a wireless repeater to the third node; the third node generates the control actions and translates them into ASCII commands to be sent to the ADAM-4024DAC (see Figure 12).

The DLC1248 LED dimmer pilots the LED lamp with a DC voltage ranging from 0 V (0% of the LED panel power) to 10 V (100% of the LED panel power). The control law aims to maintain the illuminance level at a value of 250 lux, which typically represents a good value to ensure visual comfort. The control law is generated by using a PID technique. The control law generated by using the rapid prototyping architecture has been implemented by using Argosd I motes [10], experimental Sky-based motes under development at Università Politecnica delle Marche, as shown in Figure 10.

**Figure 13:**
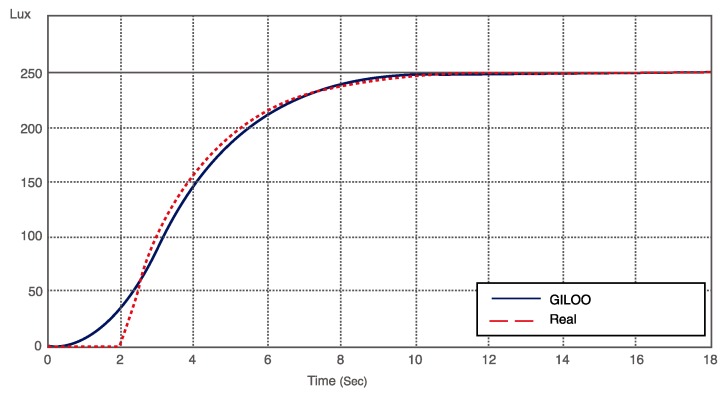
Lux level of a real experiment (dotted) and for the GILOO experiment (solid).

Figure 13 highlights the lux level set point and the comparison between the results obtained in the rapid prototyping scenario and the real scenario, where noises, disturbances and transmission delays can be introduced both by wireless communications or electronic devices.

## 7. Conclusions

In this work, the co-simulator GILOO has been designed and tested for control over a WSN. The module is based on the GISOO plugin, and in particular, it integrates LabVIEW and COOJA, jointly with the Advanced Sky software module and the Sky-DAC driver for Contiki OS. GILOO has been tested in a real CPS application consisting of an LED lighting wireless system. The proposed case study aims to emulate the typical industrial automation process architecture. Results show that GILOO allows one to design, test and debug control policies in real or simulated scenarios and, finally, to improve CPS rapid prototyping. Furthermore, GILOO permits one to analyze the system performance comparing the output between the co-simulation and the real system. Currently, the authors are considering a possible future development for the GILOO co-simulator. It is related to increasing the LabVIEW block usability, since the simulation of a large amount of nodes, within high UDP traffic messages, causes a decreasing of the speed simulation. In detail, this problem could be addressed by optimizing the developed code both for LabVIEW and COOJA and realizing packed libraries for encapsulating the functions. In any case, we are still working on this improved version, and we plan to release the entire tool in the future.

## Abbreviations

The following abbreviations are used in this manuscript:

ADC: Analog (to) Digital Converter
COOJA: COntiki OS JAva Simulator
CPS: Cyber Physical System
DAC: Digital (to) Analog Converter
FPGA: Field Programmable Gate Array
GILOO: Graphical Integration of Labview and cOOja
GUI: Graphical User Interface
HLA: High Level Architecture
LED: Light Emitting Diode
MSPSIM: MSP430 SIMulator
NCSWT: Networked Control System Wind Tunnel
NS-2: Network Simulator series 2
PiccSIM: Platform for integrated communications and control design, Simulation, Implementation and Modeling
PLC: Programmable Logic Controller
PWM: Pulse Width Modulation
SCADA: Supervisory Control And Data Acquisition
TOSSIM: TinyOS SIMulator
UART: Universal Asynchronous Receiver Transmitter
UDP: User Datagram Protocol
WCPS: Wireless Cyber-Physical Simulator
WNCS: Wireless Network Control System
WSAN: Wireless Sensor and Actor Network
WSN: Wireless Sensor Network
